# Trends in the removal of Aboriginal children in Western Australia.

**DOI:** 10.23889/ijpds.v7i3.1970

**Published:** 2022-08-25

**Authors:** Benjamin Harrap, Alison Gibberd, Melissa O'Donnell, Koen Simons, Sandra Eades

**Affiliations:** 1 The University of Melbourne; 2 University of South Australia

## Objectives

To understand in greater detail the rates of interaction Indigenous children have with the child protection system in Western Australia and how they vary by birth year, geographic region, and between family units.

## Approach

We used data linkage between the Western Australian Department of Communities (child protection data), the Midwives Notification System, and Deaths Registry. Our cohort consisted of all Aboriginal and/or Torres Strait Islander children born in Western Australia between 2000 and 2013, with child protection data available from 2000 to 2015. We used cumulative incidence curves to visualise the overall rates of interactions with the child protection system and decompose interactions using seven two-year birth cohorts, geographic child protection regions and family units.

## Results

Collaboration has been cyclical, with results from the qualitative research and discussion with the reference groups informing the initial quantitative research direction. Findings from this research were presented back to the groups, resulting in further questions and directions to explore.

The journey so far has been one of learning and understanding the skills I have and the role they can play whilst acknowledging the limits of my own knowledge and the need for Indigenous voices to guide the research in order to be doing research with Indigenous peoples, rather than on them.

## Conclusion

Co-design with Indigenous peoples is critical for doing research which affects them or uses data from their communities. Understanding my own cultural background and acknowledging the limitations of my experience continues to be important for honest and meaningful collaboration.

